# Introgression Breeding in Cowpea [*Vigna unguiculata* (L.) Walp.]

**DOI:** 10.3389/fpls.2020.567425

**Published:** 2020-09-16

**Authors:** Ousmane Boukar, Michael Abberton, Olaniyi Oyatomi, Abou Togola, Leena Tripathi, Christian Fatokun

**Affiliations:** ^1^Cowpea Breeding Unit, International Institute of Tropical Agriculture, Kano, Nigeria; ^2^Genetic Resources Center, International Institute of Tropical Agriculture, Ibadan, Nigeria; ^3^Biosciences, International Institute of Tropical Agriculture, Nairobi, Kenya

**Keywords:** cowpea, *Vigna unguiculata*, crop wild relatives, introgression, genetic diversity, genomics, new plant breeding techniques

## Abstract

The narrow base of genetic diversity characteristic of cowpea can be attributed to it being self-pollinating, evolving from narrow wild germplasm and exhibiting very limited gene flow between wild and cultivated types. Backcrossing to introduce simply inherited desirable traits and utilization of improved breeding lines and varieties as parents in crossing programs further narrowed the genetic base of cowpea varieties. In most cowpea breeding programs, genes for resistance and market traits were pyramided into lines characterized by high levels of acceptance to farmers and consumers. Besides predisposing widely distributed improved varieties to genetic vulnerability, a narrow base of genetic variation may be contributing to the plateauing in cowpea grain yield, which compromises genetic gains. Cross compatible wild relatives have not been used in variety development because breeders shy away from them due to their tiny seed size, unattractive seed coat color and texture, pod shattering, and susceptibility to viruses. A number of wild cowpea relatives, both within and outside section *Catiang* of *Vigna* species, have been evaluated for their reaction to cowpea insect pests and diseases. *Vigna vexillata* lines were resistant to the legume pod borer (*Maruca vitrata*), the cowpea weevil (*Callosobruchus maculatus*), and *Striga gesnerioides* but are cross incompatible with cultivated cowpea. Some lines among the cross compatible wild relative *V. unguiculata* ssp. *dekindtiana* were found to be resistant to aphid in the seedling stage, while others showed good levels of drought and heat tolerance. Molecular markers are being generated to identify quantitative trait loci (QTL) with effects on some desirable attributes in cowpea. Modern breeding tools, including transgenics, can be applied for the improvement of cowpea, bypassing the natural barriers of traditional breeding. Transgenic cowpea with *Bt* gene *cry1Ab* showing resistance to *M. vitrata* has been released in Nigeria. Genome editing, a powerful emerging tool, can also be used for developing improved cowpea varieties with durable resistance to pests and diseases.

## Introduction

Cowpea, also known as black-eyed pea, belongs to section *Catiang* (DC) Verdc. genus *Vigna*, tribe *Phaseoleae* in Family *Fabaceae* (Maréchal et al., 1978). It is a self-pollinating diploid with chromosome number 2n = 22 and a genome size of about 613 (Arumuganathan and Earle, 1991) to 640.6 Mb (Lonardi et al., 2019). It is cultivated worldwide especially in Africa, Central and South America, Asia, Oceania, Southern Europe, and USA (Quin, 1997) while most is produced in the dry savannah regions of sub-Saharan Africa (SSA) in companion with mainly sorghum and millet in the same fields (Steele, 1972). Only a small proportion of SSA farmers grow cowpea as a sole crop (Steiner, 1982). In comparison to many other crops, cowpea is more adapted to drought stress and even performs relatively better in depauperized soils that are characteristic of the agro-ecologies where the crop is most extensively grown in SSA (Mortimore et al., 1997). In addition, being a legume, it has the capacity of fixing atmospheric nitrogen, some of which it utilizes for growth and development, while some do remain in the soil for the benefit of following crops (Quin, 1997). The protein rich grains are commonly eaten across the regions in different food dishes while the leaves are consumed as a pot herb, especially in East Africa. Farmers in the dry savannah areas feed their livestock with cowpea haulm, which has high nutritional value. Many efforts were devoted previously to developing cowpea varieties with high grain yield, while in recent times attention is being focused on developing dual purpose varieties with both high grain and fodder yields. It was reported that in four years, 75% of farmers in Kano State, Northern Nigeria adopted a dual-purpose cowpea variety (IT89KD-288) due to its additional fodder yield (Inaizumi et al., 1999). Many of the farmers’ traditional varieties (local varieties) show attributes similar to dual purpose types, but the former are generally more adapted to intercropping. Progress can be made through breeding to increase both grain and fodder yields simultaneously because grain and fodder yields in cowpea tend to be positively correlated (Samireddypalle et al., 2017). Highly significant variations were also detected among cowpea lines for measured livestock nutrition traits such as nitrogen (N), ﬁbre fractions, *invitro* digestibility, and metabolizable energy content. The authors also reported the absence of any trade-offs between grain yield and haulm fodder quality traits. Interestingly, haulm acid detergent lignin, and grain yield were observed to be inversely correlated, suggesting that high grain yielding varieties had lower haulm lignin content.

The center of diversity for cultivated cowpea is reported to be in West and Central Africa while that for wild relatives is in southern parts of Africa. Wild cowpea relatives are mostly distributed from Namibia through Botswana, Zambia, Zimbabwe, Mozambique, South Africa, and Swaziland (Padulosi and Ng, 1997). These authors based their suggestion on primitive characteristics such as perenniality, small seed size, pod shattering, hairiness of plant parts, distinct exine on pollen surface and outcrossing among other traits that are associated with wild relatives. They further suggested that the Transvaal Region of South Africa represents the center of speciation of *Vigna unguiculata* since the most primitive forms of the wild relatives, especially varieties *rhomboidea*, *protracta*, *tenuis*, and *stenophylla*, all in section *Catiang* are mostly found there. The variety *rhomboidea* has, the narrowest range of geographical distribution, mainly in the area from 20°S to 27°S and 26°E to 32°E and an isolated presence around Cape Town in South Africa. The wild relatives of cowpea include *Vigna unguiculata* subsp. *dekindtiana*, *V. unguiculata* subsp. *stenophylla*, *V. unguiculata* subsp. *tenuis*, *V. nervosa*, *V. vexillata*, *V. oblongifolia*, *V. frutescens*, *V. reticulata*, *V. luteola*, *V. pygmaea*, *V. gazensis*, and *V. nuda* (Padulosi and Ng, 1990). Samples of each of these wild cowpea relatives were collected from different parts of Zimbabwe. In addition, samples of *V. kirkii*, *V. platyloba*, and *V. wittei* were collected from Tanzania, further showing that the center of diversity of wild cowpea is in the southern parts of Africa. Therefore, collections made from this sub-region should, to a large extent, represent the most significant diversity among wild cowpea relatives.

The center of domestication for cultivated cowpea is still to be agreed among taxonomists. Some suggested that the area of cultivated cowpea domestication is located from Senegal in West Africa to Eritrea in the East (Kouam et al., 2012). Following a single-nucleotide polymorphism (SNP) marker analysis carried out on 1,200 cowpea lines, two gene pools were identified, one each in West Africa and in East Africa (Huynh et al., 2013). The authors concluded that cowpea has dual domestication. Another molecular marker diversity study also confirmed West and Central Africa as the region of cowpea domestication (Xiong et al., 2016). The authors suggested that the yard-long-bean, *V. unguiculata* ssp. *sesquipedalis* and *V. unguiculata* ssp. *biflora* evolved in India and south East Asia after cowpea was introduced into the region from Africa. The domestication of cowpea experienced a double bottleneck (Pasquet, 1996). The first was from its wild progenitor, which resulted in primitive cultivar group cv.-gr. Biflora and cv.-gr. Textilis and the second from the primitive cultivar-group to the evolved cultivar-groups cv.-gr. Melanophthalmus in West Africa and cv.-gr. Sesquipedalis in Asia. However, the generally accepted immediate progenitor of cowpea is *Vigna unguiculata* ssp. *dekindtiana*, which is widely distributed across Africa (Padulosi and Ng, 1997). This wide distribution of the immediate progenitor across the region may contribute to the lack of clarity as to the exact location of the domestication of cultivated cowpea. The oldest evidence so far is from archaeological excavations made in Ghana, in the West Africa sub-region, which suggest that cowpea domestication took place before 1500 BC (D’Andrea et al., 2007).

Despite the availability of a fairly large number of cowpea germplasm being conserved in some gene banks, the genetic base of the crop remains narrow. An evaluation of genetic diversity among improved cowpea varieties and breeding lines obtained from IITA breeding nursery using simple sequence repeat (SSR) markers revealed that improved cowpea varieties, in general, have a narrow genetic base (Li et al., 2001). This can be attributed to breeders’ consistently using improved elite lines as parents in crosses to generate segregating populations in their programs. In addition, the backcross method of breeding is often used to introduce simply inherited traits to varieties as a means of correcting some of the weaknesses that may be present in existing varieties. Being a highly self-pollinated crop evolved from a single wild progenitor also contributed to its narrow genetic base. In order to broaden cowpea’s genetic base, it is necessary to utilize alien germplasm, especially from among cross compatible wild relatives.

Genetically modified (GM) cowpea is being developed at some research stations in SSA (ACB, 2015). The currently available GM cowpea variety carries an insecticidal *Cry1Ab* gene encoding a *Bacillus thuringiensis* (*Bt*) toxin. Ghana, Malawi, Burkina Faso, and Nigeria are the countries where confined field evaluations of transgenic *Bt* cowpea lines have been performed (Togola et al., 2017). Genetic engineering was undertaken for the development of cowpea with resistance to the legume pod borer *Maruca vitrata* Fabricius (Lepidoptera: Crambidae), the most damaging and economically important post-flowering insect pest of cowpea in SSA, following the inability to successfully cross cowpea with those of its wild relatives that exhibit resistance to this pest (Togola et al., 2017). The first successful report of an insertion of a transgene into cowpea through transgenesis involved the *Cry1Ab Bt* gene (Popelka et al., 2006). The *Bt* gene has now been transferred through backcrossing to some improved and released cowpea varieties in four SSA countries. The transgenic *Bt* cowpea lines, when evaluated in the field, showed high levels of resistance to *Maruca* as the larvae which are responsible for causing damages on cowpea flowers, pods, seeds, and young shoots were killed following their feeding on the plant (Mohammed et al., 2014). One major limitation of the *Bt* genes is their poor expression in higher eukaryotes (Bett et al., 2017), and a second limitation is their selective properties that target mostly Lepidopteran species. This implies that cowpea farmers who adopt these transgenic varieties will still need to protect their crops against other insect pests that are not controlled by *Bt* gene.

The first GM insect resistant cowpea variety [*SAMPEA 20-T*, Pod Borer Resistant (PBR) Cowpea] has recently been approved for commercialization in Nigeria (Crop Biotech, 2019). This commercialized variety has protection against *Maruca vitrata*, a pest that can cause grain yield losses of up to 60% (Singh et al., 1990). The new variety was developed by scientists at the Institute for Agricultural Research (IAR), Ahmadu Bello University, Zaria Nigeria, in collaboration with several partners under the coordination of the African Agricultural Technology Foundation (AATF) (Mohammed et al., 2014). Although the PBR cowpea confers resistance to *M. vitrata*, it is still susceptible to other insect pests, which are capable of causing major problems to cowpea cultivation. The crop is attacked at every stage in its life cycle by different species of insects, each capable of causing significant grain yield losses. The most damaging insect pests of cowpea, in addition to the pod borer are the cowpea aphid (*Aphis craccivora* Koch), flower bud thrips (*Megalurothrips sjostedti* Trybom), and pod sucking bugs (*Clavigralla tomentosicollis* Stål, *Riptortus dentipes* Fabricius) and storage pests such as bruchid (*Callosobruchus maculatus* Fabricius and *Bruchidius atrolineatus* Pic) (Jackai and Singh, 1988). These pests have devastating effects on cowpea production in the field and seeds in storage. Insect pest control in cowpea in SSA farmers’ fields remains abysmally low due mainly to high costs of synthetic insecticides that are imported. This has continued to depress the productivity of improved cowpea varieties being released in several countries. Therefore, there is a need to generate broad-spectrum resistance to these pests by stacking pest resistance genes through application of the transgenic approach or editing of the host plant genome. In this paper, we review studies carried out to identify wild cowpea relatives with traits such as resistance to insect pests, *Striga*, drought tolerance and high nutritional quality that could be exploited in the development of new improved cowpea varieties and some other potential traits that could be introgressed into cowpea as well as efforts made to introgress desirable traits from wild to improved cultivars.

## Exploring the Diversity and Potential of CWRs for Introgression Breeding

The value of conserved genetic resources depends largely on the uniqueness of the samples in the collection and the extent of the diversity captured (Upadhyaya et al., 2006). The wild relatives of cowpea represent valuable genepools yet to be tapped for cowpea variety development. Wild relatives of cowpea contain genotypes that independently evolved within specific environments and are potentially crucial in cowpea genetic improvement in the context of climate change. The success of a plant-breeding program depends largely on the availability of a large germplasm collection, representing the genetic diversity of the crop species and the knowledge of important morphological and agronomic features of the accessions in the collection. For a long-term crop improvement program, a large and diverse germplasm collection is an invaluable source of supply of parental strains for hybridization and subsequent development of improved varieties (Chheda and Fatokun, 1982). Since its establishment in 1967, the International Institute of Tropical Agriculture (IITA) has accumulated a large collection of cowpea germplasm exceeding 15,000 accessions of cultivated varieties drawn from over 100 countries and over 1,500 accessions of wild *Vigna* species. This collection contains cowpea genotypes that display variations in many traits such as plant pigmentation, plant type, plant height, leaf type, growth habit, photo-sensitivity or insensitivity, maturity, nitrogen fixation, fodder quality, heat and drought tolerance, root architecture, pod traits, seed traits, grain quality and resistance to major diseases, root-knot nematodes, insect pests (aphids, bruchid, thrips), and parasitic weeds (*Striga* and *Alectra*). Wild forms and closely related species of cowpea, therefore, could have potential as additional sources of useful genes for cowpea improvement (Baudoin and Marechal, 1985; Padulosi and Ng, 1990).

Characterization of germplasm accessions was largely concerned with the description of accession composition and morphological characteristics or phenotypic expressions (Longwe, 1996). This characterization process involves recording those characters, which are heritable and visible by observation as expressed in any environment. This type of characterization is an account of the plant morphology, either throughout its life cycle or only at maturity. In more recent times and with the advent of molecular markers, it has become a common practice to use markers for characterization of crops’ germplasms. These markers are available in abundant numbers, neutral to environmental influence and, therefore, more robust in discriminating among germplasm lines. The molecular markers that have been used in cowpea characterization include restriction fragment length polymorphism (Fatokun et al., 1993), amplified fragment length polymorphism (Coulibaly et al., 2002), random amplified polymorphic DNA (Ba et al., 2004), SSRs (Li et al., 2001), inter SSR analysis (Ajibade et al., 2000), gene derived markers (Wang et al., 2008), and SNPs (Xiong et al., 2016).

Studies reporting the use of wild cowpea relatives in the development of improved varieties are rare to come by, if any. Breeders have shied away from utilizing wild relatives in cowpea variety development because of undesirable attributes. Generally, wild cowpea relatives have small seed size, unattractive seed coat color and texture, tendency to be susceptible to several virus diseases, pod shattering, weedy, and indeterminate growth habit (Rawal et al., 1976) among the cross compatible ones. With the genomic tools being developed for cowpea, breeders should be more inclined to embark on pre-breeding activities to make good progress by exploiting the potential benefits of wild cowpea relatives. Small seed size, a characteristic of wild cowpea relatives, appears to be dominant to large size (Rawal et al., 1976). In SSA, the larger the size of the cowpea grain the higher is the consumer preference; hence, breeders place some emphasis on seed size while making selections. The gene action on seed size in cowpea is mostly additive but with significant additive x additive epistatic effects and conditioned by at least eight effective factors (Drabo et al., 1984). The possibility of linkage drag of some undesirable traits that are characteristic of the wild relatives can now be readily eliminated by applying molecular tools.

It is interesting to note that two QTLs for the number of seeds per pod were found in cowpea on chromosomes Vu05 and Vu09 (Lo et al., 2018). The allele *CSp09* on chromosome Vu09, which accounted for 21.09% of the variation for number of seeds per pod, was donated by the wild parent. A transcription factor identified in the region of the QTL *CSp09* was found in Arabidopsis to be associated with ovule development (Wei et al., 2015). A higher number of seeds per pod should result in higher grain yield as have been reported in soybean (Van roekel and Purcell, 2016), peanut (Songsri et al., 2009), and rapeseed (Yang et al., 2016). Hence, the generally small seed size of the wild cowpea relatives notwithstanding, they are capable of contributing to higher grain yield in cultivated cowpea through an increase in the number of seeds in each pod, i.e., increasing the number of ovaries per pod. The QTLs associated with this trait suggest that the number of seeds per pod is heritable and can, therefore, be selected for in breeding of cowpea for higher grain yield. Long peduncles enable plants to carry their pods above the canopy thereby helping to reduce the amount of damage caused by *Maruca vitrata* (Oghiakhe et al., 2011), and allowing easier harvesting of pods. they are also a source of fibre (Pasquet, 1996). This trait is present in the wild cowpea line used in generating the linkage map. A single QTL on chromosome VU05 that explains 71.83% of phenotypic variation was detected (Lo et al., 2018). Understanding the genetic basis of perenniality should facilitate success in perennializing cultivated cowpea, which could result in the development of new potentially higher-yielding varieties.

## Useful Traits Present in Some Wild Cowpea Relatives

Efforts have been made to identify wild cowpea relatives, which exhibit traits that are desirable and could be transferred to improved cowpea varieties. Some of the traits detected following these efforts are discussed below:

### Insect Resistance

Aphid (*Aphis craccivora* Koch) is the first major insect pest that affects cowpea at an early developmental stage. It attacks seedlings by sucking sap from the plant, especially when drought occurs. Seedlings can be killed if the infestation is high and accompanied by delayed rainfall (Singh and Jackai, 1985). Until recently, a dominant gene (*Rac*) detected in a germplasm line (TVu-3000) conferred resistance to this pest and the gene was incorporated in breeding lines developed at IITA (Bata et al., 1987). However, this gene has become ineffective and all plants having the gene now succumb to the insect, thus calling for the detection of other sources of aphid resistance genes. A cowpea wild relative, TVNu-1158, showed resistance to aphid in the seedling stage (Souleymane et al., 2013). This wild relative has been successfully crossed to cowpea, and a set of RILs developed, which have been used to generate a linkage map of cowpea. In addition, QTLs with effects on domestication related traits have been detected using this RIL population (Lo et al., 2018). The set of RILs have been evaluated for resistance to aphid, and some of them were found to be resistant. These are now being used as parents in a crossing program to some elite lines with the aim of transferring the resistance to the latter. In a recent study, three cultivated cowpea accessions TVu-6464, TVu-1583, and TVu-15445 with good levels of resistance to *A. craccivora* comparable to the level found in an existing resistant TVu-801 were reported (Togola et al., 2020). These new sources of aphid resistance in both wild and cultivated cowpea lines need to be tested for allelism. Should all or some of them turn out to be non-allelic, pyramiding them in good genetic backgrounds would lead to the development of new resistant cowpea varieties that will be resilient to aphid. In addition, the resistance will remain present in such varieties over a longer period. The resistance mechanism in the three accessions listed above was established to be linked to their low sucrose levels and high levels of kaempferol and quercetin (aglycones of phenolic compounds) (Togola et al., 2020). A flavonoid HPLC fingerprint carried out on some wild and cultivated cowpea also showed a positive relationship between aphid resistance and high levels of flavonoid glycosides (Lattanzio et al., 1997).

Some lines of *Vigna vexillata* have been reported to exhibit resistance to the legume pod borer (*Maruca vitrata*), the most cosmopolitan of cowpea insect pests capable of reducing grain yield by up to 60% (Singh et al., 1990). It is the cause of severe damages to pods, seeds, and young tender plant tissues. All of five accessions of *V. vexillata*, 10 of *V. oblongifolia*, two of *V. macrosperma*, and one *V. reticulata*, all wild cowpea relatives, were completely resistant to Maruca (Singh et al., 1990). Trichomes present on the pods of two *V. vexillata* accessions (TVNu-72 and TVNu-73) were partly responsible for their resistance to *Maruca*, although when trichomes were removed the insect’s larvae developed but not optimally (Jackai and Oghiakhe, 2009). On the other hand, the two lines were resistant to the pod sucking bug, *Clavigralla tomentosicollis* Stål (Hemiptera, Coreidae) as the insect’s nymphs could not survive whether trichomes were present or removed. However, adult pod sucking bugs’ feeding damage score was slightly higher when trichomes were removed from the pods. They concluded that the mechanisms of insect resistance present in the two *V. vexillata* lines were antibiosis and antixenosis. All accessions of *V. luteola* (3), *V. vexillata* (17), *V. macrosperma* (2), and *V. angustifolia* (3), were resistant to *Clavigralla tomentosicollis* Stål (Singh et al., 1990). All six and 27 accessions of *V. luteola* and *V. vexillata*, respectively, showed resistance to the cowpea seed weevil, *C. maculatus* Fabricius (Singh et al., 1990).

### Striga and Alectra Resistance

*Striga gesnerioides* [Wild.] Vatke and *Alectra vogelii* [Benth], members of the family Scrophulariaceae (Kuijt, 1969), are parasitic weeds that attack cowpea plants in the field. They are root pathogens with *Striga* alone capable of causing up to 50% (Parker, 1991) or even 83% to 100% (Emechebe et al., 1991; Cardwell and Lane, 1995) yield loss in cowpea. A single *Striga* plant can produce a large quantity of seeds (up to 90,000), many of which may remain viable in the soil for up to 15 to 20 years (Parker, 1991). *Striga*, which is generally more virulent, is predominantly present in the dry savannah (Sudan and Sahel) agroecologies where most cowpea is produced, while *Alectra* is found in the moist savanna areas such as the guinea savannah. The development of varieties that are resistant to these parasites is the most efficient way of controlling them in SSA farmers’ fields. Genes that confer resistance to both parasites have been identified and incorporated into improved varieties that farmers are growing in different countries. A major constraint to disseminating resistant cowpea varieties across the region is the existence of many races of *Striga* present in the different countries. Five races were initially identified (Lane et al., 1997). Race 1 is found in Burkina Faso, race 2 in Mali, race 3 in Nigeria and Niger, race 4 in the Republic of Benin, and race 5 in Cameroon. Three of these races (1, 3, and 5) were later reported to be present in Nigeria (Singh and Emechebe, 1997). Two previously unknown races parasitizing cowpea have since been reported one each in Senegal—race 6 and Zakpota in the Republic of Benin—SG4z (Botanga and Timko, 2006) making the number of identified races seven. The *Striga* resistance genes *Rsg-1*, *Rsg-2*, and *Rsg-3* were obtained from two different cowpea lines (Fery and Singh, 1997). The cowpea line B301 is the source of two duplicate genes *Rsg-1* and *Rsg-2*. Some wild cowpea relatives were evaluated for their reactions to the parasite in highly *Striga* infested fields at Minjibir near Kano in northern Nigeria located in the Sudan savannah agroecology and some of them showed high levels of resistance. Among the wild cowpea relatives that showed immunity to *Striga* are TVNu-1070, TVNu-1083, TVNu-585, TVNu-1535, TVNu-1537, TVNu-1647, and TVNu-491 belonging to the following *Vigna* species: *ambacensis*, *parkeri*, *oblongifolia*, and *reticulata* (Oyatomi et al., 2016). Only one *Striga* resistant accession, TVNu-1589, among the tested wild relatives belong to section *Catiang*. *Striga* seeds cannot be moved across national boundaries due to plant quarantine regulations but seeds of resistant cultivars and cross compatible wild relatives can be shared with cowpea breeders across the West African sub-region for evaluation against the different races present in their respective countries. Two duplicate genes with symbols *Rav-1* and *Rav-2* control resistance of cowpea to *Alectra* (Singh et al., 1993). Genes conferring resistance to *Striga* and *Alectra* are neither allelic nor linked (Atokple et al., 1995). However, additional sources of resistance genes could be present in the resistant wild cowpea relatives and these should boost resistance levels if non-allelic to any of the reported dominant resistance genes that have been incorporated into improved varieties. The resistance genes present in these wild cowpea relatives could serve as additional sources of resistance to potentially new *Striga* races that may develop as a result of climate change effects.

### Nutritional Qualities

Eight wild *Vigna* s pp. (*V. vexillata*, *V. vexillata macrosperma*, *V. luteola*, *V. oblongifolia*, *V. unguiculata dekindtiana*, *V. racemosa*, *V. reticulata*, and *V. ambacensis*) were evaluated for chemical characteristics such as protein, amino acid profiles, starch digestibility as well as for anti-metabolic compounds, such as trypsin inhibitors, cysteine proteinase inhibitors, lectins, phytic acid, and tannins (Marconi et al., 1997). The aim was to identify potentially useful materials for improving the nutritional and insect resistance aspects of cowpea. *V. vexillata* showed a high protein content of up to 29.3% in the grains, while all the accessions contained high sulphur amino acids as a result of which they all showed high chemical scores. Starch content in their grains ranged from 64% to 75%. There was wide variability found in the levels of trypsin inhibitors, tannins, and lectins in the grains. Also, *V. luteola* contained high levels of these compounds, while *V. reticulata* and *V. ambacensis* as well as the immediate progenitor of cultivated cowpea, *V. unguiculata dekindtiana*, had very low levels. Significant positive correlations were found between bruchid resistance and trypsin inhibitor, tannin, and starch content. Despite the high protein content in grains of wild cowpea relatives, it was observed that its digestibility is low compared with cultivated lines (Marconi et al., 1990). These authors also reported that protein availability was slightly higher in wild relatives than in tested cultivated lines.

Some of the wild *Vigna* species can be utilized by humans in different ways besides the protein rich grains. For example, the tuberous roots of *V. vexillata* and *V. lobatofolia* which contain higher protein content than potato by up to 15% and six times the level found in cassava are eaten in several communities (Wehmeyer et al., 1969; Chandel et al., 1972). Some wild cowpea relatives exhibit traits that could be useful in enhancing the food value of cowpea, such as higher protein levels in grains.

### Drought Tolerance

When compared with many other crops, cowpea performs relatively better under drought conditions. However, the occurrence of drought, especially from seedling to flowering stage can still have adverse effects on its productivity. The existing level of drought tolerance in improved varieties can be enhanced through breeding. The habitats from where some of the accessions of the wild relatives were collected, and their growth habits, suggest that some of them could be potential sources of genes for drought tolerance. For example, accessions of the subspecies *tenuis* and *stenophylla* were collected mostly in the dry savannah agro-ecologies of Mozambique and Zimbabwe where the soils are generally sandy (Padulosi and Ng, 1997). It is conceivable that some of the wild cowpea relatives collected from these dry environments should be potential sources of drought tolerance genes. This is because they have become adapted to such environments. A recent study on Mesoamerican common bean showed that wild relatives of the crop collected from dry areas of South America are found to be good sources of drought tolerance (Teran et al., 2020). Some wild cowpea lines are characterized by perennial growth habit and this attribute could also contribute to drought tolerance as they retain their greenness, and are able to survive from one cropping season to another through the intervening dry season.

### Longevity

Cultivated cowpea is an annual crop with improved varieties being either extra early, i.e., maturing in 60 days, early (65–75 days), medium (75–100 days), or late (more than 100 days). Most of the traditional farmers’ varieties are, on the other hand, late maturing types. These latter types also tend to spread, cover the ground quickly (Rawal, 1975), and are day length sensitive (Craufurd et al., 1997). Farmers who prefer to grow cowpea as a sole crop usually choose day neutral, extra early or early maturing varieties while those who grow cowpea in intercrop prefer the dual purpose and late maturing types. In the dry savannah regions of SSA, cowpea fodder is appreciated by livestock farmers because of its relatively high nutritional quality. It has been observed that in the dry savannah agro-ecologies, cowpea farmers make a reasonable income from sales of cowpea fodder (Inaizumi et al., 1999; Samireddypalle et al., 2017). Even after insect pests such as pod borer and pod sucking bugs have damaged pods and seeds on the crop while growing in the field, farmers are still able to harvest the cowpea fodder for sale to livestock owners. Cut and carry systems of cowpea fodder are well developed in Asia and Australia where yields can reach up to 4 tons per hectare (Heuze et al., 2015). Some cross compatible cowpea relatives are perennial and can therefore be grown by farmers and herders as long-term sources of fodder for animals. A set of RILs generated from a bi-parental cross between cultivated and a wild cowpea relative with the latter exhibiting perennial growth habit was evaluated, and some of the RILs demonstrated longevity by surviving and staying green for more than 700 days from planting in pots (Lo et al., 2020). An understanding of the genetic basis of perenniality should facilitate efforts in perennializing cultivated cowpea which could result in the development of new potentially higher yielding varieties. Additionally, cowpea farmers in the Sahelian region who keep livestock may have a preference for such long surviving plants as they will be good sources of fodder over an extended period. Varieties with stay green characteristics over a long time could be of immense benefit to the itinerant herdsmen, who are based mainly in the dry savannah areas but migrate to the more humid coastal areas of West Africa for greener fodder during the dry season when the savannah vegetation has dried up. The availability of such varieties will also help stem the farmers/herdsmen clashes that are commonly reported from different parts of Nigeria and elsewhere in the West African sub-region.

## Interspecific Hybridization and Backcross Breeding: Barriers and Overcoming Them

Some traits desired in improved cowpea varieties are present in several of the crop’s wild relatives. For example, genes for resistance to the legume pod borer (*Maruca vitrata*) and pod sucking bugs were found in accessions of *V. oblongifolia* and *V. vexillata* (IITA, 1988; Singh et al., 1990). Among the wild cowpea relatives outside of section *Catiang*, *V. vexillata*, which belongs to section *Plectrotropis*, was reported to be the most phylogenetically close to cowpea based on RFLP analysis (Fatokun et al., 1993). This notwithstanding all attempts made to cross cowpea with *V. vexillata* failed to produce any interspecific hybrid (Fatokun, 2002). Embryological studies were carried out to determine the causes of interspecies incompatibility between the two (Barone and Ng, 1990). According to the authors, incompatibility results from lack of fertilization in most instances and the collapse of ovules following pollination. Several crosses were made between cowpea and *V. vexillata* with the aim of transferring the desirable genes for resistance to insect pests such as pod borer, flower bud thrips, and seed storage weevil from the wild to cultivated genotype. Some of the efforts made to overcome the cross incompatibility between cowpea and *V. vexillata* (Fatokun, 2002) were as follows:

- Making crosses among several accessions of both species. This was attempted from the recognition that certain cross combinations are more productive than others. In some crop species such as *Nicotiana* s pp., it was observed that crosses between some accessions were more successful than others (Pittarelli and Stavely, 1975). However, in the case of cowpea and *V. vexillata*, none of the cross combinations resulted in an interspecies hybrid.
- Application of growth hormones such as 2,4-D and NAA on the pistil before or after pollination was also attempted to promote a successful interspecies cross between cowpea and *V. vexillata*. Growth hormones have been successfully used to prolong pod retention in interspecific crosses in *Phaseolus vulgaris* from 15 to 30 days (Al-Yasiri and Coyne, 1964). In the case of cowpea, spraying low concentrations of 2,4-D on flowers of *V. vexillata* before and after pollination with cowpea resulted in pod retention until pod maturity. However, no viable hybrid seed was obtained from the retained pods (Fatokun, 2002).
- Rescue of embryos extracted from ovules retained for up to 4 days following interspecific cross pollination—*V. vexillata* x *V. unguiculata* ssp. *unguiculata* was attempted. The embryo and endosperm obtained following crosses between *V. vexillata* and cowpea collapsed between five to eight days after pollination (Barone et al., 1992). Embryos were found to form and developed up to the globular stage, after which further development stopped when cowpea was crossed with *V. vexillata* (Fatokun, 1991). Placing these interspecific embryos in MS culture media containing growth hormones did not result in further development (Fatokun, 2002).
- Polyploidization of both species prior to being used for making interspecific hybridizations was carried out. Both cowpea and *vexillata* are diploids with a chromosome number of 2n = 22. Only accessions of cultivated cowpea responded positively to treatments with low concentrations of colchicine. However, the polyploid cowpea lines generated were fertile and set seeds, still could not produce any hybrid when crosses were made between them and *V. vexillata* in both directions, i.e., using the polyploid as male and female (Fatokun, 2002).
- The use of a parthenocarpic cowpea line (R1 36) obtained from the University of California Riverside as a female parent to crosses with accessions of *V. vexillata* was attempted. However, no successful hybrid resulted (Fatokun, 2002).

From the foregoing listed unsuccessful attempts to cross cowpea *V. vexillata*, there is a very strong cross incompatibility between cowpea and its wild relatives outside the section *Catiang*. This strong cross incompatibility barrier has so far prevented the introgression of useful genes in *V. vexillata* to cowpea for variety development.

The wide crosses that have so far succeeded with cowpea are those involving members of section *Catiang* (DC) Verdc., which seem to contain the primary and secondary gene pools for cowpea. Unlike what has been observed among African *Vigna* species, successful interspecific hybridizations have been reported among Asiatic *Vigna* species that belong to section *Ceratotropis* (Piper) Verdc. Successful crosses were made between *V. radiata* x *V. dalzelliana*, *V. radiata* x *V. radiata* var. *sublobata*, *V. radiata* x *V. mungo* var. *silvestris*, and *V. umbellata* x *V. radiata* all of which are Asiatic *Vigna* species (Pandiyan et al., 2010). The successful interspecific crosses among the Asiatic *Vigna* species have enhanced genetic diversity and made it possible to take advantage of some attributes in the wild relatives for the development of high performing improved mungbean (*V. radiata*) varieties.

## Development of Populations With Introgressions From CWRs: Introgression Lines, Chromosome Substitution Lines, Advanced Backcrosses, and Others

Successful crosses between cowpea and its wild relatives have only been reported when the crosses involve those belonging to section *Catiang*. Even with some accessions from this section, it was necessary to carry out embryo rescue to be able to successfully cross some cowpea *Vigna unguiculata* ssp. *unguiculata* lines with *Vigna unguiculata* ssp. *pubescens* (Fatokun and Singh, 1987). The F_1_ plants derived from this cross exhibited partial sterility due to a low number of fertile pollen grains. In common bean (*Phaseolus vulgaris* L.), a grain legume with 11 pairs of chromosomes and a member of *Phaseolinaea* as cowpea, inbred populations were developed from three wild x domesticated backcrosses (Teran et al., 2020). The BC_1_S_4_ populations were evaluated in the fields located at three environments comprising two fully irrigated trials during two cropping seasons and an imposed terminal drought in the second season. The study revealed that the two populations derived from wild parents obtained from lower rainfall regions produced lines that gave higher yield compared to the domesticated parent in the three environments. They further reported that 20 QTLs for yield were detected in 13 regions on eight of common bean’s 11 chromosomes. Five of the QTLs showed at least one wild allele that contributed to increased yield compared to the domesticated parent. In cowpea, an advanced backcross has been generated from the cross between an improved variety (IT99K-573-1-1) and a wild relative (TVNu-1158). The wild relative was identified as an aphid resistant line (Souleymane et al., 2013). The F_1_ was backcrossed to the improved variety, and the BC_1_F_1_ further advanced to F3 generation. This population has been further advanced but have not been phenotyped.

## Characterization and Evaluation of Populations With Introgressions From CWRs for Simple and Complex Traits

A set of recombinant inbred lines (RILs) was generated from a cross between a cultivated and a wild cowpea relative. The set of RILs has been evaluated for nine different traits, including those related to cowpea domestication such as pod shattering, 100-seed weight, pod size, and flower characteristics. In addition, the RILs were genotyped using the Cowpea iSelect Consortium Array, which assays 51,128 SNPs. The 17,739 of these SNPs that passed quality controls were used to develop a high-density linkage map of cowpea (Lo et al., 2018). Sixteen quantitative trait loci (QTL) were detected across seven chromosomes for the traits measured. One major finding reported by these authors is the co-location of QTL for four traits controlling increased organ size, which are very important during the crop’s domestication process, namely, primary leaf length and width, 100-seed weight, and pod length in the same region of chromosome *Vu08*. Increased leaf size should result in a higher amount of photosynthate, which would support an increase in biomass and subsequently higher grain yields. In *Vigna umbellata* (rice bean), QTL for leaf size was closely located to those controlling seed and pod size related traits (Isemura et al., 2010). A study carried out in cowpea showed that two major QTLs are associated with flowering in the wide cross with the wild parent conferring late flowering on some RILs (Lo et al., 2018). The main QTL for the number of days from sowing to flowering in their study is located on chromosome Vu09. Using the same set of RILs, the authors reported the detection of three important loci for plant longevity and flower scent in cowpea (Lo et al., 2020). The QTL for plant longevity is located on chromosome number Vu08, the same chromosome on which some organ size related QTL were reported in their earlier study. Within this perenniality QTL region, they observed the presence of genes encoding an F-box protein (*Vigun08g215300*) and two kinases (*Vigun08g217000*, *Vigun08g217800*), both involved in physiological processes that include flowering time regulation and plant longevity. QTL for flower scent, an important trait in insect induced cross pollination, was detected on two chromosomes, Vu01 and Vu11.

## “Omics” Applications to Introgression Breeding

Compared with many other crops, the development and application of genomic tools for cowpea improvement has lagged behind and only a few relevant studies have been reported. Some progress has been recorded in recent times following identification of molecular markers associated with some important traits in the crop, but marker application in variety development is still very limited. In the Republic of Niger, marker assisted backcross was used to transfer *Striga* resistance gene from the breeding line IT93K-693-2 into three farmers’ preferred varieties, *viz*., IT90K-372-1-2, KVx30-309-6G, and TN5-78 (Salifou et al., 2016). The microsatellite marker SSR1 was used to track and introgress the resistance gene in the segregating populations. Marker-assisted breeding based on SNP genotyping was used to stack large seed haplotypes into a CB27 background with 22 g/100 seeds using a rare haplotype associated with large seeds at the *Css-1* locus from cowpea variety IT82E-18 (18.5 g/100 seeds) (Lucas et al., 2015). These authors used foreground and background selections during two cycles of backcrossing based on genome-wide SNP markers and obtained families with very large seeds (28–35 g/100 seeds). Three major QTLs associated with bacterial blight, one on Vu09 (*qtlblb‐1*), and two on Vu04 (*qtlblb‐2* and *qtlblb‐3*), accounting for 30.58%, 10.77%, and 10.63% phenotypic variations, respectively, have been identified (Dinesh et al., 2016). They successfully introgressed the QTL on Vu09 from cultivar V‐16 into the bacterial leaf blight susceptible variety C‐152 through marker‐assisted backcrossing (MABC). These are the first reports on the application of marker assisted breeding in cowpea improvement. With these demonstrated successful introgression of desired genes into cowpea from cultivated lines, breeders can now apply marker assisted technology in exploiting the hidden potentials present in wild cross compatible cowpea wild relatives. Carrying out genome-wide association studies (GWAS) using wild crop relatives can be a good and successful starting point for identifying homologous genes in other species belonging or not to the same botanical family (Huard-chauveau et al., 2013). Cowpea can also benefit from utilizing its cross compatible wild relatives as exemplified in the example of common bean mentioned above (Teran et al., 2020).

## Gene Editing of COWPEA to Facilitate Their Use in Breeding

Genome-editing is a new breeding tool that enables the efficient and precise targeted modification of plant genome and therefore has a lot of potential for crop improvement. It has been applied in a wide variety of plant species for functional gene analysis and the improvement of economically important traits. Several tools like Zinc Finger Nucleases (ZFNs), TAL effector proteins (TALENs), RNA-guided nucleases (RGENs), and CRISPR (clustered regularly interspaced short palindromic repeats)/Cas9 (CRISPR associated protein 9) have been applied for improvements of crops through targeted genome editing. All these methods are based on the formation of double-strand break (DSB) at specific loci and triggering DNA repair mechanisms (Weithal and Gurel, 2016). CRISPR/Cas9 has rapidly become the most popular of the genome engineering approaches as it is comparatively simple and easy to adapt. CRISPR/Cas9 technology has been successfully applied in several plant species such as Arabidopsis, *Nicotiana banthemiana*, rice, wheat, maize, sorghum, tomato, soybean, apple, citrus, poplar, and coffee (Song et al., 2016; Scheben et al., 2017; Breitler et al., 2018). Recently, the CRISPR/Cas9-based genome editing system was developed for cowpea, demonstrating the disruption of the endogenous representative symbiotic nitrogen fixation (SNF) gene (Ji et al., 2019).

Genome editing has enormous potential for improving agronomic traits of crops such as resistance to diseases and insect-pests. Resistance to diseases and insect-pests in cowpea crop can be induced using genome editing technique following several approaches such as knocking down of the susceptibility genes, manipulating the effector-target interaction, modifying the receptor gene to boost the host immune system and/or altering the plant hormones responsible for antagonistic action of defence leading to enhanced broad-spectrum resistance (Bisht et al., 2019). Genome editing can be applied to both cultivated and wild cowpea relatives following which any useful traits exhibited by the modified lines can be introgressed in the breeding programs.

## Conclusions

The wild relatives of cowpea abound in the southern parts of Africa, where available evidence suggest their center of diversity exists. Seeds of a number of these wild relatives have been collected and conserved in the genetic resources center at IITA Ibadan, Nigeria, and elsewhere. Breeders, plant physiologists, and entomologists, in particular, have evaluated some of the wild cowpea relatives for the purpose of identifying those with the potential to contribute genes for resistance to insect pests and drought tolerance in cultivated cowpea. These efforts have led to the discovery of accessions of wild relatives that show resistance to insect pests of cowpea. For example, accessions of *V. oblongifolia* and *V. vexillata* that were evaluated showed resistance to insect pests such as aphid, flower bud thrips, pod borers, and bruchids. However, very strong cross incompatibility exists between cultivated cowpea and *vexillata* and this has made it impossible for breeders to introgress the resistance genes into the former. However, few cross compatible wild cowpea relatives show resistance to some constraints, and these wild relatives are being used as sources of resistance genes. The development of molecular markers in cowpea will help breeders utilize these cross compatible wild relatives in cowpea variety development. Undesirable traits such as small seed size, unattractive seed coat color, etc. present in the wild relatives can be more readily selected using molecular marker technologies. The new breeding technologies will be of immense benefit while introgressing additional traits such as perennial growth habit and stay green characteristics existing in wild relatives into cultivated cowpea.

## Author Contributions

OB led several of the studies reported here and wrote most of the sections of the paper with strong contributions of CF who also designed the paper and is the corresponding author. MA and OO shared the wild accessions from the IITA Genetic Resources Centre and conducted the screening of wild relatives for *Striga r*esistance. LT wrote the section on the genetic engineering and gene editing. AT wrote the sections on insect pests. All authors contributed to the article and approved the submitted version.

## Conflict of Interest

The authors declare that the research was conducted in the absence of any commercial or financial relationships that could be construed as a potential conflict of interest.
